# A Systematic Review of Laparoscopic Ultrasonography During Laparoscopic Cholecystectomy

**DOI:** 10.7759/cureus.51192

**Published:** 2023-12-27

**Authors:** Bakhtawar Awan, Mohamed Elsaigh, Mohamed Marzouk, Azka Sohail, Beshoy Effat Elkomos, Ahmad Asqalan, Safa O Baqar, Noha Elgndy, Omnia Saleh, Justyna Szul, Anna San Juan, Mohamed Alasmar

**Affiliations:** 1 General and Emergency Surgery, Northwick Park Hospital, London, GBR; 2 Thoracic Surgery, Norfolk and Norwich University Hospital, Norwich, GBR; 3 Colorectal Surgery, Derriford Hospital, University Hospitals Plymouth, Plymouth, GBR; 4 Acute and Emergency Medicine, Frimley Park Hospital, Surrey, GBR; 5 General and Gastrointestinal Surgery, Laboratory for Surgical and Metabolic Research, Brigham and Women’s Hospital, Harvard Medical School, Boston, USA; 6 General Surgery, Salford Royal Hospital, University of Manchester, Manchester, GBR

**Keywords:** laparoscopic ultrasonography, bile duct stones, choledocholithiasis, bile duct injury, laparoscopic cholecystectomy, intraoperative ultrasound

## Abstract

We aim to investigate the potential of laparoscopic ultrasonography (LUS) as a replacement for intraoperative cholangiography (IOC) in the context of laparoscopic cholecystectomy focusing on various aspects related to both techniques. We made our search through PubMed, Web of Science, Cochrane Library, and Scopus, with the use of the following search strategy: (“laparoscopic ultrasonography” OR LUS OR “laparoscopic US” OR “laparoscopic ultrasound”) AND (“laparoscopic cholecystectomy” OR LC). We incorporated diverse studies that addressed our topic, offering data on the identification of biliary anatomy and variations, the utilization of laparoscopic ultrasound in cholecystitis, the detection of common bile duct stones, and the criteria utilized to assess the accuracy of LUS. A total of 1526 articles were screened and only 20 were finally included. This systematic review assessed LUS and IOC techniques in cholecystectomy. IOC showed higher failure rates due to common duct catheterization challenges, while LUS had lower failure rates, often linked to factors like steatosis. Cost-effectiveness comparisons favored LUS over IOC, potentially saving patients money. LUS procedures were quicker due to real-time imaging, while IOC required more time and personnel. Bile duct injuries were discussed, highlighting LUS limitations in atypical anatomies. LUS aided in diagnosing crucial conditions, emphasizing its relevance post surgery. Surgeon experience significantly impacted outcomes, regardless of the technique. A previous study discussed that LUS's learning curve was steeper than IOC's, with proficient LUS users adjusting practices and using IOC selectively. Highlighting LUS's benefits and limitations in cholecystectomy, we stress its value in complex anatomical situations. LUS confirms no common bile duct stones, avoiding cannulation. LUS and IOC equally detect common bile duct stones and visualize the biliary tree. LUS offers safety, speed, cost-effectiveness, and unlimited use. Despite the associated expenses and learning curve, the enduring benefits of using advanced probes in LUS imaging suggest that it could surpass traditional IOC. The validation of this potential advancement relies heavily on incorporating modern probe studies. Our study could contribute to the medical literature by evaluating their clinical validity, safety, cost-effectiveness, learning curve, patient outcomes, technological advancements, and potential impact on guidelines and recommendations for clinical professionals.

## Introduction and background

In the past decades, laparoscopic ultrasonography (LUS) has been integrated into clinical use to address the constraints of minimally invasive surgical procedures [1,2], as one or few small incisions are made in the abdominal wall and thin tubes are inserted with a camera targeting a specific place to give real-time ultrasound imaging for the internal organs [3]. The technique employs the same entry points as a regular laparoscopic cholecystectomy (LC). A flexible ultrasound transducer is introduced through the umbilical port, while the endoscope is inserted through an epigastric location just superior to the umbilicus. The common bile duct (CBD) is located by scanning the liver and positioning it on the inner side of the gallbladder. The gallbladder is gently moved upwards, and the ultrasound device is placed right over the CBD [4,5]. By doing this, the junction where the right and left hepatic ducts meet, along with the connection of the cystic duct (CD), becomes visible [6]. After that, the path of the CBD toward the duodenum is traced, and by adjusting the tip of the device, a side-to-side view of the CBD can be seen [7].

On the other hand, intraoperative cholangiography (IOC) is a medical procedure that is done during surgery, particularly in the context of gallbladder and biliary tract surgeries [8]. It involves the use of a contrast medium (dye) injected directly into the biliary system, which includes the CBD and its associated structures, such as CD, gallbladder, pancreatic duct, and duodenum. The contrast medium helps visualize the anatomy of the bile ducts through X-ray imaging, allowing surgeons to assess the presence of any abnormalities, such as gallstones, strictures, or anatomical variations [9]. Although the use of IOC is more invasive and complicated when compared with LUS, IOC is more used in LC [10].

LC is the most common hepatic-biliary pancreatic (HPB) surgery [11]. LC is generally considered a procedure with a relatively straightforward and safe execution, provided that a comprehensive understanding of the biliary duct is ensured. In fact, thorough outlining and careful assessment of the biliary system play a pivotal role in identifying common bile duct stones (CBDS) and mitigating the risk of bile duct injury (BDI), so LC is considered the gold standard for the laparoscopic approach in comparison with open cholecystectomy [12]. During the operation of LC, one of the major risks is BDI, which is mainly caused due to the poor visibility of the biliary tract or due to the low skill of the operating surgeon [13,14]. In clinical practice, IOC holds the primary position for evaluating the structure of the bile duct and diagnosing CBDS. A significant discussion surrounds the efficacy of this procedure in either preventing or enhancing the early identification of BDI [14,15]. This matter of contention significantly impacts patients and their subsequent medical care, leading to uncertainty regarding whether IOC should be employed routinely or employed with more discretion [16,17].

IOC can have a variable challenge during the whole procedure; first of all, the cost of the IOC procedure and the length of the operation time [13,16,18], IOC into standard practice presents a set of notable hurdles. From a practical standpoint, these challenges encompass the requirement for preliminary dissection before IOC, a step that can prove to be technically demanding, particularly in cases involving acute or chronic inflammatory conditions [19]. Additionally, there is the intricacy of cannulating a CD that may be inherently short, thin, or even tortuous in its anatomical orientation. Overcoming these technical obstacles effectively becomes imperative for the successful execution of IOC and achieving accurate biliary visualization [18].

In contrast to IOC, LUS is characterized as a more minimally invasive option, offering quicker execution, cost-effectiveness, absence of reported adverse effects, and the capability for repeated utilization throughout the surgical procedure without exposing the patient to radiation. This makes it a notably more desirable choice, especially for pregnant, elderly patients, patients with more cosmetic concerns, pediatric patients, diabetic patients, patients with obesity, and high malignancy patients [20-22]. However, despite these advantages, the routine application of LUS in the context of LC is extremely rare when compared to the prevalence of IOC. Merely 1% of surgeons have adopted this technique into their practice [21]. Many previous reports discussed the importance and benefits of LUS over the last period [23-30]. We aim to compare LUS and IOC from various points of view to gain a comprehensive understanding of the comparative effectiveness between LUS and IOC. It is crucial to thoroughly examine parameters such as the failure rate and underlying causes, cost-effectiveness considerations, incidence and nature of BDIs, duration and speed of the surgical operation, the impact of surgeon experience on outcomes, and the likelihood and significance of incidental findings during both LUS and IOC procedures.

## Review

Methods

The study was designed according to the Cochrane Handbook for Systematic Reviews of Interventions and reported under the Preferred Reporting Items for Systematic Reviews and Meta-Analyses (PRISMA) guidelines [31,32].

Literature Search

We made our search through four different databases, including PubMed, Web of Science, Cochrane Library, and Scopus, with the use of the following search strategy: (“laparoscopic ultrasonography” OR LUS OR “laparoscopic US” OR “laparoscopic ultrasound”) AND (“laparoscopic cholecystectomy” OR LC).

Studies Selection and Eligibility Criteria

We considered all the studies that met our inclusion criteria whether they were randomized controlled trials (RCTs), non-RCTs, cross-sectional, or cohort.

Population: Include participants whether males or females without any restrictions to age, who are undergoing LC for choledocholithiasis, involve prospective data collection, compare IOC or LUS to the gold standard method, and attempt both LUS or IOC on all patients.

Intervention: Laparoscopic ultrasonography was the intervention used.

Outcomes: The outcomes included cost-effectiveness, speed of operation, biliary duct injury, surgeon experience, and incidental findings. Two distinct authors conducted relevance screening by evaluating titles and abstracts, followed by complete texts.

Quality and Risk of Bias

The methodological quality of the studies was evaluated using the assessment tool provided by the National Institutes of Health (NIH) [33]. The author's evaluation of the studies is categorized into "good," "fair," or "poor" based on the scores acquired during the assessment process.

Data Extraction

We extracted the data into Excel sheets (Microsoft Corporation, Redmond, WA). The extracted data contained the following items: (1) summary characteristics, including study ID, site, study design, sensitivity, positive predictive value, negative predictive value inclusion criteria, and conclusion; (2) baseline data, including study arms and sample size; (3) outcomes, including cost-effectiveness, speed of operation, biliary duct injury, surgeon experience, and incidental findings.

Results

Literature Search Results

**Figure 1:**
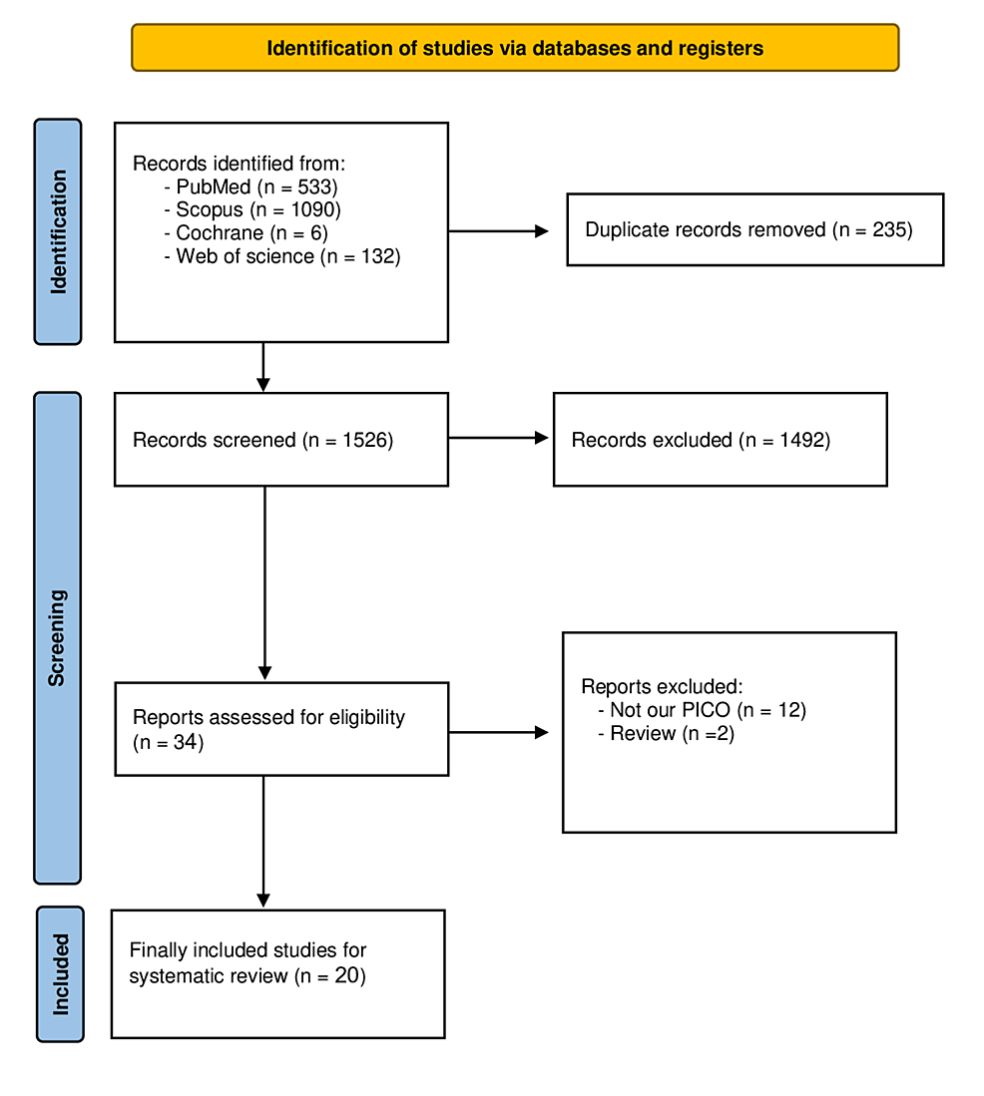
PRISMA flow diagram PRISMA: Preferred Reporting Items for Systematic Reviews and Meta-Analyses.

Our search was made until the 25th of June 2023. A total of 1526 results were obtained, and after the removal of duplicates and applying our inclusion and exclusion criteria, finally, 20 [2,7,19,33-49] papers were included in our systematic review (Figure 1).

Study Characteristics and Quality and Risk of Bias

We included 20 studies with a total sample of 9189 patients and the specific details of the summary and baseline characteristics are shown in Table 1. All the studies that we included had fair quality except for three studies that had good quality according to the NIH tool. Specific details of the NIH assessment are described in Table A1 in the Appendix.

**Table 1:** Summary of the results and baseline characteristics of the included studies ASA: American Society of Anesthesiologists; BD: bile duct; BDI: bile duct injury; CBD: common bile duct; CBDE: common bile duct exploration; CBDI: common bile duct injury; CBDS: common bile duct stone; CD: cystic duct; FIOC: fluoroscopic intraoperative cholangiography; IOC: intraoperative cholangiography; IOUS: intraoperative laparoscopic ultrasonography; LC: laparoscopic cholecystectomy; LICU: laparoscopic intracorporeal ultrasound; LOS: length of stay; LUS: laparoscopic ultrasound; OC: open cholecystectomy; TAU: transabdominal ultrasound; US: ultrasound.

Study ID	Study arms	Sample size	Study design	Age, mean ± SD (in years)	Male, N (%)	Sensitivity	Specificity	Positive predictive value	Negative predictive value	Results	Conclusion
Biffl et al. (2001) [48]	Without LUS	594	Prospective cohort	36.3 ± 0.6	98 (16%)	-	-	-	-	During the study period, 842 LCs were attempted. Patient age (37 ± 1 years) and gender (85% female) did not differ between the groups. In the US group, more patients had acute cholecystitis (p = 0.05). More LCs were performed per year by non-US surgeons than US surgeons (45 versus 37). Despite this, all bile duct complications occurred in non-US cases (2.5% overall): five CBD injuries (0.8%), six bile leaks (1%), and four retained CBD stones (0.7%). In the subgroup of patients with acute cholecystitis, there were fewer conversions to OC in the US compared with non-US cases (24% versus 36%, p = 0.09).	"IOUS is noninvasive, fast, repeatable, and can corroborate real-time visualization of the operative field. We have found that LC with IOUS is associated with fewer bile duct complications (CBD injuries, bile leaks, and retained CBD stones) than LC without adjunctive imaging. The success rate of LC in cases of acute cholecystitis is slightly higher when IOUS is used as an aid to dissection. In the absence of definitive prospective data, we recommend routine use of IOUS when performing LC, particularly in patients with acute cholecystitis."
	LUS	248		37.2 ± 1.0	30 (12%)	100	96.23	-	-		
Catheline et al. (2002) [49]	LUS	900	Prospective cohort	-	-	80	99	89	99	Laparoscopic ultrasonography was performed in all 900 patients. Cholangiography was performed in 762 (85%). The mean (range) duration was 9.8 (4–21) minutes for laparoscopic ultrasonography and 17.6 (7–42) minutes for cholangiography. For the detection of common bile duct stones, with a kappa coefficient of 0.57 (95% confidence interval (CI) 0.43 to 0.71), the non-random concordance between the two methods was considered to be fair to good. The sensitivity of laparoscopic ultrasonography was 0.80 (95% CI: 0.65 to 0.91) and its specificity was 0.99 (95% CI: 0.98 to 1.00). The respective values for cholangiography were 0.75 (95% CI: 0.59 to 0.87) and 0.99 (95% CI: 0.98 to 1.00). The examinations combined had a sensitivity of 0.95 (95% CI: 0.86 to 0.99) and a specificity of 0.98 (95% CI: 0.96 to 1.00).	"Laparoscopic ultrasonography and intraoperative cholangiography are complementary, as the combination of both methods maximizes the intraoperative detection of choledocholithiasis."
	IOC	900		-	-	75	99	79	98		
Elkerkary et al. (2021) [50]	LUS	53	Cross-sectional	41 ± 9.3	17 (32.07%)	80	95.83	66.67	97.87	"Our study enrolled 53 patients. No intraoperative complications occurred in all enrolled patients. LUS was successful in all 53 (100%) cases, while IOC was successful in 50 (94.3%) cases. IOC had an accuracy rate of 100% (50 patients) in defining biliary ducts at the porta hepatis compared to 84.91% (45 patients) for LUS with a failure rate of 15.09% (p = 0.60). Concerning stones detection, LUS accuracy indexes were as follows: sensitivity = 80%; specificity = 95.83%; positive predictive value (PPV) = 66.67%; negative predictive value (NPV) = 97.87% 99; and diagnostic odds ratio (DOR) = 92. IOC accuracy indexes were as follows: sensitivity = 80%; specificity = 93.33%; PPV = 57.14%; NPV = 90%; and DOR = 56."	"The results of the current study encourage using IOC as an effective, accurate, feasible, and safe technique to visualize the biliary tree while performing LC."
	IOC	53				80	93.33	57.14	90		
Deziel (2022) [34]	LUS	732	Prospective cohort	-	527 (72%)	-	-	-	-	"LUS demonstrated the cystic duct–common bile duct junction, the common hepatic duct, the common bile duct to the ampulla, and the right hepatic artery in 95.8% of cases. Among 56 of 111 (50%) patients in group I for whom initial dissection failed to result in adequate anatomic identification, subsequent LUS provided sufficient anatomic identification to allow completion of a laparoscopic operation in 87.5%. Group I patients were more likely to have acute cholecystitis (p < 0.0001) and Tokyo Guidelines 2018 grade II or III acute cholecystitis (p < 0.001). LUS changed operative management for 19 of 256 (7.5%) Group II patients and 10 of 361 (2.8%) Group III patients by demonstrating common bile duct stones that resulted in common bile duct exploration with stone clearance. Five patients had common bile duct stones that were not detected by LUS. There were no major bile duct or vascular injuries."	"The primary value of LUS during LC is for anatomic identification when there are severe local inflammatory conditions. In this setting, LUS imaging can facilitate the safe completion of LC or an early decision for an alternate operative strategy. When performed primarily for common bile duct stones or as routine practice, LUS results in CBDE for a limited proportion of patients"
Sebastian et al. (2019) [35]	LUS	126	Retrospective cohort	-	46 (36.5%)	-	-	-	-	"Laparoscopic ultrasound ensured a safe plane of dissection and no biliary or vascular complications were observed. Stent insertion into the common bile duct before the operation undoubtedly made the identification of anatomical structures easier. Conversion to an open procedure was deemed necessary in only 6 patients (4.8%)."	"Laparoscopic ultrasound facilitates the successful performance of LCs. It can be used at any time during the operation; it is noninvasive; and there is no need to use X-rays or contrast dye, or to cannulate the cystic duct. The most important advantage of LUS is that it leads to a lower number of conversions and intraoperative complications by identifying anatomical relationships in the plane of dissection."
Sebastian et al. (2020) [36]	LUS	156	Cross-sectional	48.43 ± 14.6	-	-	-	-	-	"The identification rate of ultrasonographic landmarks – the upper border of “Mickey Mouse” sign (MMS) (the equivalent of the Rouviere’s sulcus), the bile duct, and the hepatic artery – was significantly higher in patients with the body mass index ≥ 30 kg/m2 and fibrosis and chronic inflammation in the gallbladder neck than B-SAFE. LUS was also significantly more successful in the identification of the bile duct in the whole study group than B-SAFE. There were no significant differences according to the identification of the duodenum. The conversion rate was 2.6%, and we did not observe any BDI."	"Visual landmarks defined in B-SAFE are not as reliable as ultrasonographic landmarks; thus, LUS should be taken into consideration in the first place as a method of navigation around the gallbladder."
Gwinn et al. (2013) [37]	LUS	44	Prospective cohort	55.5	18 (40.9%)	-	-	-	-	LUS identified the extrahepatic bile ducts in all cases. Of the cases, 40 (91%) were completed laparoscopically. OC patients had a higher rate of acute cholecystitis and preoperative percutaneous cholecystostomy tubes and a higher mean ASA classification. Intraoperatively, LC patients had significantly less estimated blood loss and fewer drains were placed. Postoperatively, LC patients had significantly fewer total complications, Clavien-Dindo grade 3 complications, biliary complications, biliary reinterventions, intra-abdominal abscesses, and bleeding complications. LC patients had significantly fewer ICU admissions and shorter LOS.	"By allowing identification of the extrahepatic bile ducts during difficult cholecystectomy, LUS results in a high rate of successful laparoscopic completions. Laparoscopic cholecystectomy is associated with better clinical outcomes than OC for patients with severe cholecystitis."
Hakamada et al. (2008) [38]	Before routine IOUS	368	Retrospective cohort	53.3 ± 13.9	140 (38.04%)	-	-	-	-	IOUS was highly feasible even in patients with high-grade cholecystitis. No BDI was observed after the introduction of the educational program, despite 72% of operations being performed by inexperienced surgeons. Incidences of other morbidity, mortality, and late complications were comparable before and after the introduction of routine IOUS. However, the operation time was significantly extended after the educational program began (p < 0.001), and the grade of laparoscopic cholecystitis (p = 0.002), use of IOUS (p = 0.01), and the experience of the surgeons (p = 0.05) were significant factors for extending the length of operation.	"IOUS during LC was found to be a highly feasible modality, which provided accurate, real-time information about the biliary structures. The educational program using IOUS is expected to minimize the incidence of BDI following LC, especially when performed by less-skilled surgeons."
	Routine IOUS	276		57.1 ± 13.6	107 (38.8%)	76	99	-	-		
Halpin et al. (2002) [39]	Fluoroscopic intraoperative cholangiography	400	Prospective cohort	48.8 ± 0.8	100 (25%)	-	-	-	-	Demographics and preoperative diagnoses were similar in the two groups. Excluding those who were converted to open cholecystectomy and those in whom an imaging exam was not attempted, FIOC was successful in 361 of 374 (97%) patients and LICU was successful in 377 of 380 (99%) patients (p < 0.03). The mean times (±SEM) to complete FIOC and LICU were 16.0 (±0.5) min and 5.1 (±0.1) min (p < 0.0001), respectively. Choledocholithiasis was detected in 25 patients (7%) undergoing FIOC and in 39 patients (10%) undergoing LICU (p 4 0.1). During LICU the common bile duct was visualized in continuity from the cystic duct to ampulla in 90% of cases. The common bile duct could not be completely visualized in continuity at the middle or distal portion of the common bile duct in 5% and 6% of LICU cases, respectively. One LICU patient (0.3%) with an incompletely visualized duct had a suspected stone confirmed by postoperative endoscopic retrograde cholangiopancreatography (ERCP). One patient with negative FIOC (0.3%) had a retained stone treated by postoperative ERCP.	"LICU is safe and accurate, and it permits a more rapid evaluation of bile duct stones than FIOC during laparoscopic cholecystectomy. The false-negative rate of both imaging techniques is less than 1%."
	LUS	394		48.1 ± 0.7	87 (22%)	97.5	100	-	-		
Hublet et al. (2009) [21]	IOC	695	Prospective cohort	-	-	96.9	99.2	86	99.8	A prospective database was permitted to evaluate the results of the two methods in 968 consecutive cholecystectomies. Nine hundred and twenty-five were performed by laparoscopy, 18 (1.9%) by laparotomy, and 25 (2.6) necessitated a conversion. The systematic use of the IOC was gradually replaced by a systematic use of the LUS. The success in delineating and evaluating the CBD, the detection of a CBDS, and any type of bile duct complication, especially of CBDI, were registered. All the CBDS suspected by LUS were controlled by IOC. The patients were followed for 1 and 6 months. Six hundred and eighty-five IOC and 269 LUS were performed. The procedure was technically unsuccessful in 35 IOC (5.1%) (mainly due to difficulty in catheterizing the cystic duct) and in 2 LUS (1%) (due to steatosis). Concerning the detection of CBDS, 31 were detected by IOC (4.5%) and 16 by LUS (6%). Five IOCs were considered as false positive, 1 as false negative (sensitivity and specificity of 96.9 and 99.2%), and 1 LUS as false positive (sensitivity and specificity of 100 and 99.6%). Five CBDI were detected in the complete seria: 2 during the dissection before the IOC, 1 thermic injury, 1 late stenosis, and 1 lateral stenosis by the cystic clip detected by LUS. However, none of these CBDI could have been prevented by IOC.	"In our experience, in this prospective study, LUS has been certainly as effective as IOC as a primary imaging technique for bile duct. It permitted to detect CBDS with a high specificity and sensitivity, and CBDS and was not followed by an increase in CBDI"
	LUS	269				100	99.6	94	100		
Kothari et al. (2013) [40]	LUS	253	Prospective cohort	43.5	61 (24%)	-	-	-	-	Two hundred and fifty-three patients were prospectively enrolled over 6 years. Seventy-six percent were female, mean age and preoperative body mass index (calculated as kg/m2) were 43.5 years and 48, respectively. The mean time to complete the LUS was 4 minutes. The mean common bile duct diameter measured 3.7 mm via LUS and 4.0 mm via TAU. Transabdominal ultrasound and LUS identified 61 and 60 patients with cholelithiasis, respectively (p = 0.763). The sensitivity and specificity of LUS for cholelithiasis were 90.2% and 97.4%. Laparoscopic ultrasound identified polyps in 41 patients, and TAU identified polyps in 6 patients, 5 of which had polyps identified on LUS as well (p < 0.001). The sensitivity and specificity of LUS for polyps were 83.3% and 85.4%.	"Laparoscopic ultrasound is equivalent to TAU in detecting cholelithiasis, however, LUS detected significantly more polyps. Intraoperative LUS is an appropriate alternative to TAU in patients undergoing laparoscopic Roux-en-Y gastric bypass."
Li et al. (2009) [32]	LUS and IOC	103	Prospective cohort	-	-	82.1	98.7	95.8	93.7	The success rates of IOC and LUS were 91.3% and 100%, respectively, and the time required for LUS was significantly shorter (p < 0.01). The visualization of the intrapancreatic part of the CBD by IOC (97.3%) was significantly higher than by LUS (73.8%). The sensitivities, specificities, accuracies, positive and negative predictive values, and positive and negative likelihood ratios identifying occult CBD stones were 75.0%, 98.7%, 92.2%, 95.5%, 91.4%, 57.7, and 0.253 by IOC, and 82.1%, 98.7%, 94.2%, 95.8%, 93.7%, 63.2, and 0.181 by LUS, respectively. The McNemar test showed no significant difference between the two methods. The sensitivity of IOC combined with LUS was 92.9%, which was greater than that of IOC and LUS taken separately.	"LUS is usually performed in cases where IOC has failed or is contraindicated. The combination of both methods maximizes the intraoperative detection of occult CBD stones and should at least be recommended as two complementary methods."
Machi et al. (2009) [42]	LUS	1,381	Retrospective cohort	-	426 (30.8%)	98.6	99.56	-	-	In five centers, 1,381 patients underwent LC with LUS. LUS was successful to delineate and evaluate the BD in 1,352 patients (98.0%), although it was unsuccessful or incomplete in 29 patients (2.0%). LUS was considered remarkably valuable to safely complete LC, avoiding conversion to open, in 81 patients (5.9%). The use of intraoperative cholangiography (IOC) varied depending on the centers; IOC was performed in 504 patients (36.5%). For screening of BD stones (which was positive in 151 patients, 10.9%), LUS had a false-positive result in two patients (0.1%) and a false-negative result in five patients (0.4%). There were retained BD stones in three patients (0.2%). There were minor bile leaks from the liver bed in three patients (0.2%). However, there were no other BD injuries including BD transaction (0%). Retrospectively, IOC was deemed necessary in 25 patients (1.8%) to complete LC despite routine LUS.	"LUS can be performed successfully to delineate BD anatomy in the majority of patients. The routine use of LUS during LC has obviated major BD injury, compared to the reported rate (1 out of 200–400 LCs). LUS improves the safety of LC by clarifying anatomy and decreasing BD injury"
Machi et al. (2007) [2]	LUS	200	Prospective cohort	58.4	53 (26.5%)	95	100	-	-	For 193 (96.5%) of 200 patients, LUS was completed successfully, whereas IOC was needed for 7 patients (3.5%). Bile duct stones were identified in 20 patients (10%). For the detection of bile duct stones, LUS yielded 19 true-positive, 175 true-negative, 0 false-positive, and 1 false-negative results. It had a sensitivity of 95%, a specificity of 100%, a positive predictive value of 100%, and a negative predictive value of 99.4%. The postoperative complications included bile leaks from the liver bed in two patients and a retained bile duct stone in one patient. If IOC had been used selectively in a traditional manner based on preoperative risk factors, IOC would have been needed for 77 patients (38.5%). The total cost of LUS plus IOC for the current 200 patients was $26,256. The total estimated cost of selective IOC, if it had been performed for the 77 patients, would have been $31,416.	"Routine LUS accurately diagnosed bile duct stones and significantly reduced the need for selective IOC from a potential 38.5% to an actual 3.5% without adversely affecting the outcome of the LC or increasing the overall cost. The routine use of LUS during LC is accurate and cost-effective."
Onders et al. (2005) [43]	LUS	105	Prospective cohort	50.5	25 (24%)	100	100	-	-	During the first study period, ultrasound was used in 29% of 189 laparoscopic cholecystectomies. In 2004, ultrasound was used in 77% of 66 laparoscopic cholecystectomies. Throughout both periods, fluoroscopy was only used during 6 laparoscopic common bile duct explorations (2.4% of all cases). There were no false-positive or negative ultrasounds, and there were no bile duct injuries.	"As experience with ultrasound cholangiography increases, there is little indication for fluoroscopic cholangiography except for rare questions concerning anatomy and during therapeutic maneuvers for common bile duct stones."
Perry et al. (2008) [7]	IOC	239	Prospective cohort	-	-	NA	-	-	-	Intraoperative bile duct imaging was performed in 371 (94%) of 396 LCs performed for cholelithiasis. As recorded, IOC was performed in 239 cases, LUS in 236 cases, and both in 104 cases. Choledocholithiasis was present in 50 patients (13%). Common bile duct stones (CBDS) were identified by LUS in 3% of the patients without preoperative indicators of CBDS, and in 10% of the patients with one or more indicators. As shown by the findings, LUS had a positive predictive value of 100%, a negative predictive value of 99.6%, a sensitivity of 92.3%, and a specificity of 100% for detecting CBDS. Also, LUS identified clinically significant bile duct anatomy in 6% of the patients. In 1995, LUS was used for 20% of cases, whereas by 2005, it was used for 97% of cases. Conversely, the use of IOC decreased from 93% to 23%.	"With moderate experience, LUS can become the primary routine imaging method for evaluating the bile duct during LC. It is as reliable as IOC for detecting choledocholithiasis. In addition, LUS can locate the common bile duct during difficult dissections. Based on this experience, LUS is used currently in nearly all LCs and is the sole method for bile duct imaging in 75% of these cases. IOC is used as an adjunct to LUS when LUS imaging is inadequate, when stronger clinical indicators of choledocholithiasis are present, or when biliary anatomy remains uncertain."
	LUS	236		-	-	92.3	100	100	99.6		
Pfluke et al. (2011) [44]	LUS	50	Retrospective cohort	37.4	15 (31%)	100	100	-	-	A retrospective review was conducted of all patients who underwent cholecystectomy over 29 months. IOUS was performed after the release of the medial and lateral peritoneal attachments of the gallbladder. Of the 65 patients, 43 (66%) had an urgent operation. The mean operative time was 89.6 minutes (range 45 to 196 minutes). IOUS was used routinely, when available, in 50 patients (77%). The biliary anatomy was completely observed in 48 patients (96%). IOUS identified significant biliary abnormalities in 20 patients (40%), including the presence of a foreshortened cystic duct (CD) (< 1 cm) in 7 patients (14%), common bile duct stones in 4 patients (8%), abnormal CD anatomy in 4 patients (8%), and abnormal vascular anatomy in 8 patients (16%).	"No patient was converted to open operation, no bile duct injury occurred, and no patient required subsequent biliary intervention. IOUS is effective at observing biliary anatomy in the setting of acute disease, and may be a useful tool during these difficult cases."
Shaaban (2014) [45]	LUS	70	Retrospective cohort	63	21 (30%)	92.3	98.2	NA	-	We retrospectively studied 70 patients who underwent LUS during their laparoscopic cholecystectomy operation over 31 months. Data about the preoperative investigation, intraoperative findings, and postoperative outcomes were retrospectively collected and analyzed.	"LUS was found to be quick, safe, and effective in the intraoperative diagnosis of common bile duct stones. It does not add significantly to the operative time and is inherently safer than an intraoperative cholangiogram because it does not involve ionizing radiation. It is also more convenient, as there is no need to wear protective lead to avoid the side effects of ionizing radiation."
Tranter et al. (2001) [46]	LUS	367	Prospective cohort	52	91 (24.8%)	92	100	NA	NA	LUS visualized the CBD in 99% of patients and the common hepatic duct in 92%. It identified stones in 56 of the 61 patients with duct stones. No stones were demonstrated in the remaining 306 patients (sensitivity: 92%, specificity: 100%, positive predictive value: 100%, negative predictive value: 98%). LUS underestimated the total number of stones in 18% of patients with common duct stones. The mean common bile duct diameter was 5.0 mm before operation and 5.9 mm during the procedure in patients without duct stones, rising significantly to a mean of 9.2 mm before operation and 11.2 mm at LUS in those with duct stones (p < 0.0001).	"The combination of the demonstration of duct stones and bile duct diameter measurement makes LUS a potential replacement for IOC. Improved demonstration of the common hepatic duct would be advantageous."
Tranter et al. (2003) [47]	LUS	135	Prospective cohort	53	31 (23%)	96	100	100	98	Laparoscopic ultrasound identified the bile ducts satisfactorily in 131 cases and operative cholangiography in 121 cases. Duct stones were present in 49 cases. They were correctly identified by ultrasound in 47 cases and by cholangiography in 42 cases. There was one false positive cholangiographic examination. The sensitivity was 96% for ultrasound and 86% for cholangiography. The specificities were 100% and 99%, respectively.	"Laparoscopic ultrasound examination of the bile duct is superior to operative cholangiography and could replace it."

Failure Rate and Reasons for Failure

While performing cholecystectomy, both LUS and IOC techniques may encounter failure of the technique. The causes of technical ineffectiveness can be diverse and are contingent on factors related to both the surgeon and the patient. The lack of success in achieving desired outcomes may arise from a multitude of reasons, and these can be intricately linked to the individual skills and proficiency of the surgeon, as well as the unique characteristics and conditions of the patient. The range of factors contributing to technical unsuccessfulness is multifaceted, encompassing variables such as surgical technique, decision-making during the procedure, surgeon experience, patient anatomy, and the presence of unforeseen complications. Dili et al. [18] showed that IOC had a higher failure rate that was 5.1% due to various reasons as the difficulty of catheterization of the CD was the main reason for IOC failure, and due to the narrow lumen or the excessive inflammation of the CD or the CD valves. On the other hand, LUS showed a significantly lower failure rate with only 1% of failure of the process mainly due to steatosis. Elkerkary et al. (2021) [50] showed similar results as the LUS technique was successful in all the included patients with a 0% failure rate while IOC showed three failures out of 50 patients, a population that represents a 6% failure rate, which was due to technical failure from the operator or narrow CD or the thickness of valves at CD. Sebastian et al. [35] also showed that the failure rate while using the LUS technique is low even when used during difficult LC. Catheline et al. [49] showed a higher failure rate of the IOC procedure that reached 15%.

Cost-Effectiveness of the Technique

While the main focus when assessing the adoption of either LUS or IOC should undoubtedly be the patient’s safety and the prevention of complications, it is also essential to take into account the costs associated with these techniques as a significant secondary factor. In Machi et al.'s study [2], in 2005, the cost of LUS was put in comparison with IOC; the average cost at this period for the LUS procedure was 117 dollars while the average cost for IOC was 408 dollars, which represents a huge difference in the costs and possibility to save much money for the patient. In a study conducted by Sun et al. [51], an evaluation was carried out to assess the cost-effectiveness of utilizing LUS in comparison to IOC for the examination of asymptomatic CBDS during LC. The findings of this investigation led the authors to conclude that LUS emerges as a strategy with superior cost-effectiveness when compared to IOC. Between 2015 and 2018 in the United Kingdom, Donoghue et al. [52] found that the expense associated with utilizing LUS amounted to £183 per use, whereas the cost incurred for utilizing the IOC unit was £365 per use when examining a total of 420 patients.

Duration and Speed of Operation

Previous studies proved that LUS operation could be done faster and take a shorter time to give a real-time image, which helps to operate LC faster [38,49]. In a study conducted by Catheline et al. [49], the authors observed that the duration of LUS was approximately 9.8 minutes, contrasting with the 17.6 minutes required for IOC. It is noteworthy that implementing IOC involves the utilization of a radiological apparatus and necessitates the presence of a skilled technician to operate the equipment. Interestingly, the proficiency in employing LUS during the surgical procedure appeared to notably impact the time taken for operations involving gallstones in the gallbladder. On the other hand, Halpin et al. [39] noticed that there was no significance or difference between the two techniques when the cases were acute cholecystitis.

LUS and Biliary Duct Injury

BDI is without a doubt considered one of the most threatening morbidities that all surgeons need to avoid. The occurrence rate of BDI in LC is reported to range between 0.3% and 0.6% [53]. The more the surgeon's experience, the less the possibility of BDI occurrence. When a patient suffers from BDI, it hinders the recovery and delays the improvement of the patient in addition to the negative impact on the overall treatment. According to Deziel [34], one significant limitation of LUS in their study was its reduced effectiveness in identifying atypical anatomical arrangements within the proximal extrahepatic ducts. In instances where both LUS and IOC were conducted, IOC was found to reveal anatomical variations that were not detected by LUS in around 30% of cases. Specifically, the most common scenario in which LUS failed to identify an anatomical difference was the independent junction of the right posterior sectional hepatic duct with the common hepatic duct, as opposed to the conventional merging of the right posterior and right anterior sectional ducts to form a primary right hepatic duct. This particular anatomical variation, which is recognized in 15% to 30% of individuals, and where the CD might connect to a separate right posterior duct in 2% of individuals, is acknowledged as a potential risk factor for bile duct injury. Illustrating the application of LUS, Hublet et al. [21] showcased a case where a stenosing clip was identified through LUS, positioned dangerously near the junction connecting the CBD and the CD. This finding emerged in a patient with a regular IOC result [35]. This case serves to emphasize that common bile duct injury (CBDI) remains a possibility even after the utilization of IOC. The crucial role of LUS in this scenario was evidenced by its performance after the operation, enabling the diagnosis of CBD stenosis. Subsequent corrective measures were undertaken, effectively averting the potential occurrence of significant postoperative complications [36].

Surgeon's Experience

The surgeon's experience is an important factor in the determination of operation success. In the study conducted by Biffl et al. [48], their objective was to investigate whether the higher rate of complications observed in the non-ultrasound (non-US) group could be attributed to surgeon inexperience. The study specifically explored the relationship between surgeon expertise and the occurrence of complications. Notably, all instances in which LUS was utilized, even by surgeons without significant ultrasound experience [31], were categorized as ultrasound cases. During the study period, the non-US group's three surgeons performed a higher number of LCs per year compared to the two surgeons who usually employed LUS (45 cases per surgeon per year versus 37 cases per surgeon per year). Among the surgeons, Surgeon A, the newest member of the faculty, had the least experience. Importantly, none of the five surgeons had received specialized training in laparoscopy. Interestingly, despite the greater collective experience of the non-US group's surgeons, all instances of bile duct complications occurred within this group [38]. The quantity of LUS examinations conducted during the learning phase displays variability in different reports, with a consensus indicating that the learning curve for LUS tends to be more extensive compared to that for IOC. What becomes apparent from the review of existing literature is that surgeons who dedicate the necessary time and effort to master LUS not only develop a high level of efficiency and precision in their diagnoses but also bring about modifications in their practice [49,50,54]. These proficient surgeons opt for more judicious use of IOC, utilizing it less frequently. It is worth noting that the accuracy of LUS tends to improve in correlation with the operator's level of expertise. This underscores the importance of establishing standardized protocols for conducting LUS examinations [7,19,34,38].

The Incidental Findings During LUS and IOC

Incidental findings during LUS and IOC refer to unexpected observations or findings that are identified during these procedures but are not directly related to the primary reason for performing the procedure. These findings can encompass a wide range of conditions or abnormalities that were not initially the focus of the examination [55]. In the context of LUS, incidental findings might involve the identification of unexpected lesions, masses, or abnormalities within the organs being visualized, such as the liver, gallbladder, or nearby structures. For IOC, incidental findings could include the detection of anatomical variations, anomalies, or unexpected stones within the bile ducts that were not initially suspected or the primary target of the examination [56]. In a prospective non-randomized study by Hublet et al. [21], several notable findings were documented, including the identification of various conditions. These encompassed one instance of hemobilia (which could potentially resemble a filling defect in IOC imaging), as well as the diagnosis of a pancreatic pseudocyst, a case of intraductal papillary mucinous neoplasm, pancreas divisum, and a patient displaying microcalcifications in the pancreatic head [57]. Other authors have similarly reported the recognition of diverse conditions, such as gallbladder polyps and the presence of Mirizzi syndrome. In a study published in 2007, Machi et al. [2] conducted a prospective non-randomized investigation. During their systematic exploration through LUS, the authors managed to diagnose cystic or solid tumors situated in the liver or pancreas. This utilization of LUS enabled the surgical team to proceed with tasks such as performing biopsies, aspirations, and even conducting radiofrequency ablation for tumors in a total of six cases. Although it remains uncertain whether these tumors were coincidental findings during the perioperative period or had been diagnosed in the preoperative assessment, LUS effectively facilitated the localization of the diseases, enabling subsequent actions such as biopsies and therapeutic interventions.

Discussion

Summary of the Findings

Our paper shed light on various aspects comparing LUS and IOC in LC, including failure rate and reasons for failure in LUS and IOC during the LC. The IOC had a high failure rate compared to LUS due to various reasons and mainly due to procedure steps such as catheterization failure showed the highest incidence of failure in the IOC. The included studies in the results reveal LUS's commendable performance, characterized by a notably lower failure rate, hovering around 1%. The primary contributor to LUS failure appears to be steatosis, reflecting its robustness even in challenging surgical scenarios. In contrast, IOC exhibits a higher variability in success rates, ranging from 5.1% to 15%, as delineated by the respective studies. The elucidation of IOC failures points to a multifaceted interplay of challenges, encompassing the technical intricacies of catheterization, the restrictive nature of the narrow common duct, and the impediment posed by valve thickness. These nuanced findings collectively underscore the recurring theme of LUS's resilience and effectiveness, even in the face of anatomical and technical challenges, while concurrently emphasizing the relatively higher complexity and variability associated with IOC. This discussion enriches our understanding of the nuanced considerations surrounding the choice between LUS and IOC in clinical practice.

Cost-Effectiveness

We found that both techniques conducted at various intervals yielded congruent results, demonstrating that LUS consistently emerged as the more economical option. This financial advantage was particularly notable for patients, as the implementation of LUS translated into a reduced financial burden compared to IOC, obviating the necessity for patients to incur additional expenses. Regarding cost-effectiveness, the results that we provided in the result, which is after the year 2000, were similar to the results before the year 2000, as Falcone et al. [27] presented that the difference in the cost was significantly different between LUS and IOC, as the costs for performing IOC were almost double the costs for LUS. Additionally, as highlighted by Biffl et al. in their 2001 publication [48], the expenses associated with LUS are relatively minimal. Patients do not incur charges for the equipment, and the fact that surgeons interpret the images eliminates the need for radiologist fees. A charge of $28.25 is applied to cover the costs of the sterilization solution and the remote control cover. In contrast, IOC tends to be substantially more expensive for patients, with supplies costing over $200 and fluoroscopy expenses amounting to $2.62 per minute, alongside the anticipated radiologist fee. Regarding the LUS technique, equipment and maintenance costs are quickly offset and hospital bed days can be saved with its use. our study consistently shows LUS to be more cost-effective than IOC at various intervals, benefiting patients by reducing expenses. Post-2000 results align with earlier findings, emphasizing significant cost differences. LUS incurs minimal expenses, eliminating radiologist fees, while IOC tends to be substantially more expensive for patients. The cost savings associated with LUS extend beyond equipment, potentially reducing hospital bed days. Overall, our study supports the economic viability and favorable financial implications of choosing LUS.

Duration and Speed of Operation

The results obtained from previous studies showed a great difference in the speed of operating LC when changing the technique used as LUS would take almost half of the time that IOC requires. Additionally, patients spend less time in the hospital after LUS as it is less invasive than IOC. The collective evidence from prior studies indicates a significant disparity in the operating time for LC when transitioning from IOC to LUS. LUS consistently demonstrates a notable time-saving advantage, requiring almost half the time compared to IOC. Moreover, the less invasive nature of LUS contributes to reduced postoperative hospital stays for patients. These findings underscore the efficiency and patient-centered benefits associated with adopting LUS in the context of LC procedures.

Occurrence of BDI and Surgeon's Experience

There was no difference in the incidence of BDI between LUS and IOC and it is agreed that surgeon experience correlates with lower BDI likelihood. Finally, for the surgeons who had training on the use of LUS and who had performed LUS many times, the possibility of making mistakes is significantly lower. IOC reveals anatomical variations not detected by LUS in about 30% of cases, highlighting a potential risk for BDI. Despite a regular IOC result, LUS identified a stenosing clip near the CBD-CD junction, emphasizing the continued risk of BDI. LUS plays a crucial role in postoperative diagnosis and corrective interventions, mitigating complications.

Incidental Finding

The incidental finding during the LUS or IOC may be life-saving. Observations made during these procedures are unrelated to the primary purpose of the examination. These findings can encompass a range of conditions not initially under investigation. In the context of LUS, incidental findings may involve identifying unexpected abnormalities within visualized organs like the liver, gallbladder, or nearby structures. Having the skill to visualize the bile ducts during surgery is a crucial competence for surgeons dealing with biliary tract procedures. This imaging capability plays a key role in understanding the anatomy and identifying issues with the bile ducts. Among the methods available for real-time intraoperative imaging, IOC stands out as the most extensively practiced. Both LUS and IOC offer valuable insights, and their benefits tend to complement each other. Both LUS and IOC can reveal life-saving incidental findings. LUS excels in identifying unexpected organ abnormalities, while IOC is widely used for real-time imaging of bile ducts. Surgeons' proficiency in both techniques is crucial for biliary tract procedures, as the complementary insights provided enhance their ability to understand anatomy and identify issues during surgery. Combining LUS and IOC is key for comprehensive insights into biliary tract surgeries.

Overall Finding

Depending on the situation, one technique might prove more advantageous than the other. Additionally, another method involving near-infrared fluorocholangiography can also be used for imaging purposes [58]. Nonetheless, this approach has not been directly pitted against IOC or LUS. It is incapable of identifying CBDS and cannot visualize important blood vessels. Furthermore, its dependability across diverse clinical scenarios, particularly in the presence of evident inflammation, remains uncertain. We focused on the incidental finding as both LUS and IOC can provide additional insights into the abdominal and biliary structures, potentially leading to the recognition of conditions that might require further investigation or management. These incidental findings highlight the value of these imaging techniques beyond their primary purpose. In the year 1995, a concept known as the "critical view of safety" (CVS) was introduced. This systematic dissection technique involves carefully exposing and defining the structures within Calot's triangle, thereby establishing a clear understanding of their anatomical relationships. This approach, proposed by Strasberg et al., was put forth as a safeguard against the occurrence of BDI [59]. Yamashita et al. [60], in a study conducted in Japan, observed a reduction in BDIs after the adoption of the CVS as a dissection approach during LC. However, despite the positive outcomes reported, it remains apparent that CVS continues to be underutilized by surgeons within contemporary practice. Furthermore, there exists a suggestion that even though it has demonstrated efficacy, CVS might not be entirely adequate in fully minimizing the occurrence of CBDI. Moreover, there is an indication that CVS might not provide complete assurance in terms of effectively reducing CBDIs. This concern is supported by the observation that significant CBDIs still occur, even among surgical teams that have integrated CVS as a standardized dissection technique. This observation has been reported by de Reuver et al. [28]. When considering LUS and its role in preventing or identifying BDIs, the available data are relatively limited. Among the studies that were reviewed, instances of bile duct injuries were notably absent, with only two studies placing particular emphasis on the significance of incorporating both the CVS technique and LUS in tandem [18,40]. LUS offers the advantage of compensating for the absence of tactile palpation during LC. Moreover, it enables the examination of adjacent organs, facilitating the identification of potential underlying conditions. This includes the ability to detect liver abscesses or tumors, as well as identifying suspicious lesions in the gallbladder wall or the presence of a diverticulum in the common bile duct [24].

Strengths and Limitations

We had some strengths as the included papers have relatively high quality, and the included population is relatively high as our included papers had 9189 participants. On the other hand, one of our limitations is the lack of randomized control trials, as we included observational studies only, with high heterogeneity in the inclusion criteria, and some studies had more than one surgeon, which may result in differences related to the surgeon, and finally the absence of uniform reporting across studies may limit the ability to draw conclusive and comprehensive insights into certain aspects, highlighting the necessity for future research to explore a wider range of outcomes for a more thorough assessment.

## Conclusions

Our review is innovative and encompasses diverse outcomes, providing a comprehensive and novel exploration of LUS during LC. From our results, LUS is proved to be the first option to be used in the case of cholecystectomy and as a method of navigation around the gallbladder. As our review highlights the benefits and limitations of LUS in LC, we underscore its significance in challenging scenarios with obscured anatomy. LUS is a potentially valuable tool for confirming the absence of CBD stones, eliminating the need for biliary system cannulation. Furthermore, LUS and IOC demonstrate comparable capabilities in identifying CBD stones and visualizing the biliary tree. LUS offers safety, reduced procedure times, cost-effectiveness, and unrestricted usage. Despite initial costs and a learning curve associated with LUS, its enduring advantages remain evident. As probe technology advances, there is potential for LUS to outperform IOC. To explore this prospect further, comprehensive studies employing modern probes are essential, in addition to further investigation for the included outcomes. However, a surgeon proficient in both LUS and IOC will be well-equipped to safely manage a range of gallbladder issues.

## Appendices

Table A1

**Table 2:** ROB for cohort studies evaluated by the NIH tool ROB: risk of bias; NIH: National Institutes of Health.

Name	NIH Quality Assessment Tool for Observational Cohort and Cross-Sectional Studies															Quality rating: good (11-14 points), fair (7.5-10.5 points), or poor (0-7 points)
	1. Was the research question or objective in this paper clearly stated?	2. Was the study population clearly specified and defined?	3. Was the participation rate of eligible persons at least 50%?	4. Were all the subjects selected or recruited from the same or similar populations (including the same time period)? Were inclusion and exclusion criteria for being in the study prespecified and applied uniformly to all participants?	5. Was a sample size justification, power description, or variance and effect estimates provided?	6. For the analyses in this paper, were the exposure(s) of interest measured prior to the outcome(s) being measured?	7. Was the time frame sufficient so that one could reasonably expect to see an association between exposure and outcome if it existed?	8. For exposures that can vary in amount or level, did the study examine different levels of the exposure as related to the outcome (e.g., categories of exposure, or exposure measured as a continuous variable)?	9. Were the exposure measures (independent variables) clearly defined, valid, reliable, and implemented consistently across all study participants?	10. Was the exposure(s) assessed more than once over time?	11. Were the outcome measures (dependent variables) clearly defined, valid, reliable, and implemented consistently across all study participants?	12. Were the outcome assessors blinded to the exposure status of participants?	13. Was the loss to follow-up after baseline 20% or less?	14. Were key potential confounding variables measured and adjusted statistically for their impact on the relationship between exposure(s) and outcome(s)?	Total scores	
	Yes/No/Not reported (NR) or cannot determine (CD) or not applicable (NA)	Yes/No/Not reported (NR) or cannot determine (CD) or not applicable (NA)	Yes/No/Not reported (NR) or cannot determine (CD) or not applicable (NA)	Yes/No/Not reported (NR) or cannot determine (CD) or not applicable (NA)	Yes/No/Not reported (NR) or cannot determine (CD) or not applicable (NA)	Yes/No/Not reported (NR) or cannot determine (CD) or not applicable (NA)	Yes/No/Not reported (NR) or cannot determine (CD) or not applicable (NA)	Yes/No/Not reported (NR) or cannot determine (CD) or not applicable (NA)	Yes/No/Not reported (NR) or cannot determine (CD) or not applicable (NA)	Yes/No/Not reported (NR) or cannot determine (CD) or not applicable (NA)	Yes/No/Not reported (NR) or cannot determine (CD) or not applicable (NA)	Yes/No/Not reported (NR) or cannot determine (CD) or not applicable (NA)	Yes/No/Not reported (NR) or cannot determine (CD) or not applicable (NA)	Yes/No/Not reported (NR) or cannot determine (CD) or not applicable (NA)		
Biffl et al. (2001) [48]	Yes	Yes	Yes	Yes	NR	Yes	Yes	NR	Yes	NR	NR	NR	Yes	Yes	9	Fair
Catheline et al. (2002) [49]	Yes	Yes	Yes	Yes	Yes	Yes	Yes	NR	NR	NR	NR	NR	Yes	Yes	9	Fair
Elkerkary et al. (2021) [50]	Yes	Yes	Yes	NR	NR	Yes	NR	NR	Yes	Yes	NR	NR	Yes	NR	8	Fair
Deziel (2022) [34]	Yes	Yes	Yes	Yes	NR	Yes	Yes	NR	Yes	NR	NR	NR	Yes	Yes	9	Fair
Sebastian et al. (2019) [35]	Yes	Yes	Yes	Yes	Yes	Yes	Yes	NR	NR	NR	Yes	NR	Yes	Yes	10	Fair
Sebastian et al. (2020) [36]	Yes	Yes	Yes	Yes	NR	Yes	Yes	NR	Yes	NR	NR	NR	Yes	Yes	9	Fair
Gwinn et al. (2013) [37]	Yes	Yes	Yes	NR	No	Yes	Yes	Yes	NR	NR	Yes	NR	Yes	Yes	9.5	Fair
Hakamada et al. (2008) [38]	Yes	Yes	Yes	Yes	NR	Yes	Yes	NR	Yes	NR	Yes	NR	Yes	Yes	10	Fair
Halpin et al. (2002) [39]	Yes	Yes	Yes	Yes	Yes	Yes	Yes	NR	NR	NR	Yes	NR	Yes	Yes	10	Fair
Hublet et al. (2009) [21]	Yes	Yes	Yes	Yes	Yes	Yes	Yes	NR	Yes	NR	Yes	NR	Yes	Yes	11	Good
Kothari et al. (2013) [40]	Yes	Yes	Yes	Yes	No	Yes	Yes	NR	Yes	NR	Yes	NR	Yes	Yes	10.5	Fair
Li et al. (2009) [32]	Yes	Yes	Yes	Yes	NR	Yes	Yes	NR	Yes	NR	Yes	NR	Yes	Yes	10	Fair
Machi et al. (2009) [42]	Yes	Yes	Yes	Yes	NR	Yes	Yes	NR	Yes	NR	Yes	NR	Yes	Yes	10	Fair
Machi et al. (2007) [2]	Yes	Yes	Yes	Yes	Yes	Yes	Yes	NR	Yes	NR	Yes	NR	Yes	Yes	11	Good
Onders et al. (2005) [43]	Yes	Yes	Yes	Yes	Yes	Yes	Yes	NR	NR	NR	Yes	NR	Yes	Yes	10	Fair
Perry et al. (2008) [7]	Yes	Yes	Yes	Yes	No	Yes	Yes	NR	Yes	NR	Yes	NR	Yes	Yes	10.5	Fair
Pfluke et al. (2011) [44]	Yes	Yes	Yes	Yes	No	Yes	Yes	NR	NR	NR	Yes	NR	Yes	Yes	9.5	Fair
Shaaban (2014) [45]	Yes	Yes	Yes	Yes	No	Yes	Yes	NR	NR	NR	Yes	NR	Yes	Yes	9.5	Fair
Tranter et al. (2001) [46]	Yes	Yes	Yes	Yes	No	Yes	Yes	NR	Yes	NR	Yes	NR	Yes	Yes	10.5	Fair
Tranter et al. (2003) [47]	Yes	Yes	Yes	Yes	Yes	Yes	Yes	NR	Yes	NR	Yes	NR	Yes	Yes	11	Good
